# A Fast Reconstruction Algorithm for Fluorescence Optical Diffusion Tomography Based on Preiteration

**DOI:** 10.1155/2007/23219

**Published:** 2007-04-23

**Authors:** Xiaolei Song, Xiaoyun Xiong, Jing Bai

**Affiliations:** ^1^Department of Biomedical Engineering, Tsinghua University, Beijing 100084, China; ^2^Department 2, College of Electronic Engineering, University of Electronic Science and Technology of China, Chengdu, Sichuan 610054, China

## Abstract

Fluorescence optical diffusion tomography in the near-infrared (NIR) bandwidth is considered to be one of the most promising ways for noninvasive molecular-based imaging. Many reconstructive approaches to it utilize iterative methods for data inversion. However, they are time-consuming and they are far from meeting the real-time imaging demands. In this work, a fast preiteration algorithm based on the generalized inverse matrix is proposed. This method needs only one step of matrix-vector multiplication online, by pushing the iteration process to be executed offline. In the preiteration process, the second-order iterative format is employed to exponentially accelerate the convergence. Simulations based on an analytical diffusion model show that the distribution of fluorescent yield can be well estimated by this algorithm and the reconstructed speed is remarkably increased.

## 1. INTRODUCTION

With the discovery of biocompatible, specific fluorescent probes and the development of imaging technologies, the potential of fluorescence tomography as a means for molecularly based noninvasive imaging of biological tissues has received in recent years increased attention [1–3]. Fluorescent beacons emitting in the near-infrared (NIR) bandwidth are always preferred, since hemoglobin and water absorb minimally in this spectral window so as to allow photons to penetrate for several centimeters in tissues [4].

Using preferentially accumulated fluorescent probes as indicators or contrast agents, fluorescence optical diffusion tomography (FODT) is performed by launching light at the probes' excitation wavelength into the tissue. The fluorescent beacon absorbs the incident light, and emits light at a longer wavelength when it drops to the ground state. Then the emission is measured by an array of detection devices at the surface of the body. However, as the strong diffusion of NIR in biological tissues, reconstruction of very large unknown inside characteristics from the limited detected data at the boundary is one of the main difficulties in FODT. Many reconstructive approaches utilize iterative methods for data inversion, such as the algebraic reconstruction technique (ART) [5], Newton's or Newton-type optimization methods [6, 7], and Bayesian nonlinear least-square method [8, 9]. They are always time-consuming and far from meeting the real-time imaging demands.

In this study, a fast algorithm based on the preiteration is applied to the inversion process of fluorescence tomography. For simulating the photon's propagation in tissues with fluorescent beacons inside, a previously reported DPDW model based on Born approximation is simply introduced at the beginning. Then, the preiteration fast algorithms are presented in detail, emphasizing the second-order method. After that, the simulation using the second-order form is investigated and the results are shown. Finally, we analyze the computation burden and convergence property of the second-order iteration form and give the conclusion.

## 2. DPDW MODEL

Often a couple of diffusion equations in frequency-domain is employed to describe the propagation of both excited light and fluorescent light in diffusive medium, that is [6, 7, 10]

(1) $$\begin{array}{l} \nabla \left[D_{x}\left(\text{r}\right)\nabla {\text{Φ}}_{x}\left(r,\omega \right)\right]-\left[\mu_{a_{x}}\left(\text{r}\right)+\frac{j\omega }{c}\right]{\text{Φ}}_{x}\left(\text{r},w\right)=-\delta \left(\text{r}-{\text{r}}_{s_{k}}\right),\\ \nabla \left[D_{m}\left(\text{r}\right)\nabla {\text{Φ}}_{m}\left(\text{r},\omega \right)\right]-\left[\mu_{a_{m}}\left(\text{r}\right)+\frac{j\omega }{c}\right]{\text{Φ}}_{m}\left(\text{r},w\right)=-{\text{Φ}}_{x}\left(\text{r},\omega \right)\eta \left(\text{r}\right)\frac{1-j\omega \tau \left(\text{r}\right)}{1+{\left[\omega \tau \left(\text{r}\right)\right]}^{2}}, \end{array}$$

where Φ_*x,m*_ is the photon density for excitation (subscript *x*) or fluorescent light (subscript *m*), *D_x,m_*(**r**) is the diffusion coefficient, and *μ_a_x,m__*(**r**) is the absorption coefficient. Based on this model, the fluorescence lifetime *τ*(**r**) and the yield *η*(**r**) can be estimated through the boundary measurements. Equation (1) can be solved by analytical or numerical methods. In this paper, we use an analytical model of Born approximation for specific medium geometry to demonstrate the inversion algorithm. In fact, the fast algorithm could also be applied to arbitrary geometries, where the model is discretized by numerical methods [7, 10] or the Kirchhoff approximation [5].

In the frequency-domain model, an amplitude-modulated incident point source of photons into a diffusive medium produces a diffuse photon density wave (DPDW) [11–13]. Let an intensity-modulated point source of amplitude Θ_0_ be located at **r**_*s*_ in a homogeneous infinite medium. Then the spatial part of the originating DPDW at position **r** is [11] $U_{0}\left(r_{s}-r,k\right)={\text{Θ}}_{0}\text{exp}\left[ik\left(r_{s}-\vec{r}\right)\right]/\left[4\pi D\left(r_{s}-r\right)\right],$ with the wave number *k* = [(− *νμ_a_* + *iω*)/*D*]^1/2^, and *D* = *ν*/3*μ*′ _*s*_ is the diffusion coefficient with the reduced scattering coefficient *μ*′_*s*_ and the speed of light in the medium *ν*. Here, *ω* is the angular modulation frequency of the source. Treating fluorescent beacons as two-level quantum systems and assuming that there are no saturation or photon quenching effects, the fluorescent photon density *δu_fl_*, measured at a detector position *r_di_* due to a localized probe with volume *d* ^3^ *r_k_* embedded within the medium, is [11]

(2) $$\delta u_{fl}\left({\text{r}}_{k},{\text{r}}_{sj},{\text{r}}_{di}\right)=U_{0}\left({\text{r}}_{sj}-{\text{r}}_{k},k^{{\text{λ}}_{1}}\right)\frac{\eta \left({\text{r}}_{k}\right)}{1-i\omega \tau \left({\text{r}}_{k}\right)}\frac{\nu }{D^{{\text{λ}}_{2}}}G\left({\text{r}}_{di}-{\text{r}}_{k},k^{{\text{λ}}_{2}}\right)d^{3}r_{k},$$

with the excited source at **r**_*sj*_. Here, *λ*_1_ and *λ*_2_ represent the excited light wavelength and the fluorescent wavelength in the near-infrared section, respectively. $G\left(r_{d}-r,k^{\lambda_{2}}\right)=\text{exp}\left(ik^{\lambda 2}|r_{d}-r|\right)/4\pi |r_{d}-r|$ is Green's function solution to the diffusion equation and represents the variance of fluorescent DPDW from fluorescent probe to the detector.

For a weakly absorbing spatial distribution of fluorescent probes, the detected fluorescent DPDW at **r**_*di*_ can be found by integrating overall fluorescent sources [11, 12]. In the reconstruction, for the measurement at positions **r**_*di*_ (*i* = 1, 2, …, *M*_*i*_), the integral can be digitized as

(3) $$U_{fl}\left({\text{r}}_{sj},{\text{r}}_{di}\right)=\sum_{l=1}^{N}\delta u_{fl}\left({\text{r}}_{l},{\text{r}}_{sj},{\text{r}}_{di}\right)d^{3}r_{l}$$

due to the sources *r_sj_* (j = 1, 2, …, *M_j_*). As only one of the sources is working at a time, the total number of measurements is *M* = *M_i_* × *M_j_*. In fluorescence tomography, continuous-wave (CW) mode is always chosen, that is, *ω* = 0, and only *η* is reconstructed. Then substituting (2) in (3) will lead to the following matrix equation:

(4) *U* = *AX*,

where *U* represents an *M* × 1 column vector of the detected data, *X* is a column vector of unknown values of fluorescent yield *η* at *N* reconstructed points, and matrix *A* indicates the obtained *M* × *N* weighted coefficients.

## 3. PREITERATION INVERSE ALGORITHM

As in FODT, the inside reconstructed points number *N* is always much bigger than *M*, the measurement number at the boundary, the equation series (4) is always ill-posed and indefinite. In this case, the direct inverse matrix of *A* does not exist. However, its generalized inverse can be employed to solve (4).

### 3.1. Preiteration algorithm based on generalized inverse

If the Moore-Penrose inverse of *A* exists and is known as *A* ^+^, the unique solution of (4) which has the minimum norm and the least square can be obtained simply by [14]

(5) *X* = *A*^+^*U*.

There are several direct methods to calculate the generalized inverse *A* ^+^, for example, regularized SVD method. However, the iterative method is always preferred in computerized calculation, especially for large datasets, as it is easy to be programed and occupies much less ram than direct methods.

Supposing the residual error series $\hat{R_{k}}=I-A\hat{S_{k}}$ (*I* is the unit matrix of *M* × *M*), series

(6) $${\hat{S}}_{k+1}={\hat{S}}_{k}+S_{0}\left(I-A{\hat{S}}_{k}\right)$$

will be convergent to *A* ^+^ when *k* → ∞ [14]. Here *S*_0_ can be chosen as *αA^T^* [15], with *α* = 1/*λ*_max_. And *λ*_max_ is the maximum eigenvalue of *A* ⋅ *A* ^*T*^, where *A* ^*T*^ is the transposed matrix of *A*.

From the analysis above, a two-step reconstructed algorithm can be formed.

1. Offline preiterative step: the approximation of generalized inverse *A* ^+^ is calculated by several iterative steps of (6).
2. Online reconstruction: when the weighted matrix *A* keeps unchanged or the variation can be ignored, for updated detection *U* the unknown character *X* can be reconstructed simply through (5).

This preiteration method has already been applied to the image reconstruction in electrical impedance tomography (EIT) [15] which also belongs to the so-called “soft field” imaging as FODT, and it is proved that Landweber iteration method, which can produce higher quality reconstructed image than other direct regularized methods, is in fact a modification of the above preiteration algorithm [16]. However, compared with Landweber method, the preiteration method remarkably improves the reconstructing speed by performing the time-consuming iterative process offline.

### 3.2. Second-order iteration form

However, the first-order preiteration algorithm with form equation (6) needs the same iteration steps as the Landweber method to produce the same quality images [15]. So just like the slow convergence of Landweber, for larger-sized dataset in FODT, iteration form of (6) is also very time-consuming even in the preiteration process. In order to speed up the iteration process, the second-order iterative format

(7) $$S_{k+1}=S_{k}\left(2I-AS_{k}\right)$$

is used in our work.

To prove the convergence of the second-order form equation (7) , we examined the convergence of *S*_*k*_ and the residual error *R*_*k*_ as follows.

First, by including (7), the iterative formula of *R*_*k*_ can be obtained as

(8) $$\begin{array}{c} R_{k+1}=I-AS_{k+1}=I-AS_{k}\left(2I-AS_{k}\right)\\ ={\left(I-AS_{k}\right)}^{2}=R_{k}^{2}. \end{array}$$

Then it can be inferred that

(9) $$R_{k}=R_{k-1}^{2}=R_{k-2}^{4}=\cdots =R_{0}^{2^{k}}.$$

According to (7) and (9), *S*_*k* +1_ can be written as a function of *R*_0_ and *S*_0_ in the formula

(10) $$\begin{array}{c} S_{k+1}=S_{k}\left(I+R_{k}\right)=S_{k-1}\left(I+R_{k-1}\right)\left(I+R_{0}^{2^{k}}\right)\\ =S_{0}\left(I+R_{0}\right)\left(I+R_{0}^{2}\right)\cdots \left(I+R_{0}^{2^{k-1}}\right)\left(I+R_{0}^{2^{k}}\right)\\ =S_{0}{\left(I-R_{0}\right)}^{-1}\left(I-R_{0}^{2^{k+1}}\right), \end{array}$$

if *ρ*(*R*_0_) < 1, let k → ∞, it yields

(11) $$S_{\infty }=\underset{k\to \infty }{\text{lim}}S_{k}=S_{0}{\left(I-R_{0}\right)}^{-1},$$

where *S*_∞_ can be proved as the generalized inverse matrix of *A* [14].

However, with the first-order iteration form equation (6), residual error ${\hat{R}}_{k}=I-A{\hat{S}}_{k}$ for *k* times iteration can be expressed as

(12) $$\hat{R_{k}}=I-A\left({\hat{S}}_{k-1}+S_{0}{\hat{R}}_{k-1}\right)=\hat{R_{0}}{\hat{R}}_{k-1}.$$

Then it can be inferred that

(13) $$\hat{R_{k}}={\hat{R}}_{0}^{2}{\hat{R}}_{k-2}=\cdots ={\hat{R}}_{0}^{k+1}.$$

By comparing (9) and (13), the difference between the convergence speed of the two iteration forms can be found. If the same value of *S*_0_ is selected, *S*_k_ can be directly obtained in the *k*th step *via* the second-order form as (7) while it requires (2^*k*^−1)-step first-order iteration of (6).

## 4. SIMULATION AND RESULTS

The simulation in this paper is performed in CW mode (i.e., *ω* = 0) and under the assumption of homogenous and approximately infinite medium. The algorithm can also be applied to arbitrary geometries linearized by analytical approximation or finite element method.

**Figure 1:**
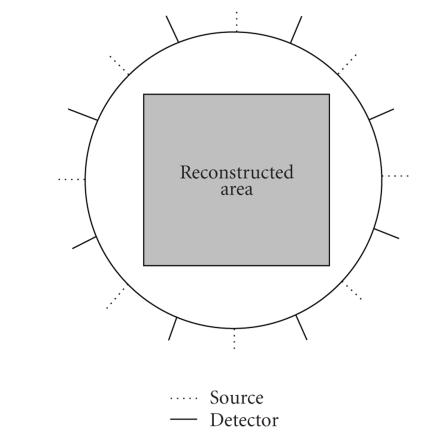
An illustration of system geometry. The excited sources and detectors are posed alternately around the circle of 50 mm diameter with equal intervals between each other. The power of the incident sources is 3 mw each.The reconstructed area is the central square slab of 0.1 cm thickness with each side of 32 mm. The solid lines represent the positions of the excited sources and the dotted lines represent the detectors.

The measurement geometry for simulations is illustrated in Figure 1. The optical properties of the media are *μ*′_*s*_ = 10 cm^−1^ and *μ_a_* = 0.03 cm^−1^ everywhere for both the excitation and emission wavelengths. The original fluorescent yield *η* is 0.05 cm^−1^ in the presence of the fluorescent probes. All the simulations were done in Matlab environment (version 7.0.1) on a 2.79 GHz Intel Pentium IV personal computer. The simulated measurement vector *U* is computer-generalized by the product of coefficient matrix *A* and the original distribution of *X*.

**Figure 2:**
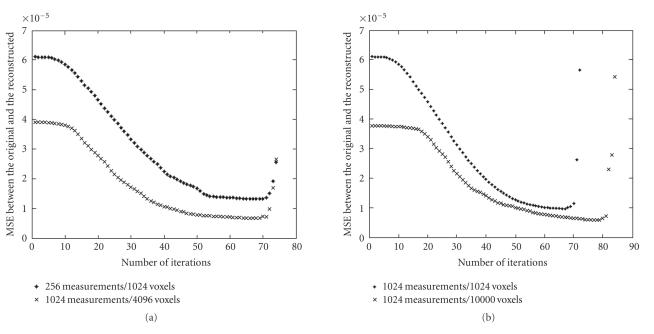
Number of iterations versus MSE (mean squared error) between the original and the reconstructed. (a) shows the MSE for datasets of 256 ∗ 1024 and 1024 ∗ 4096. Although the size is different, the iterative number where the MSE increases is the same, since they have the same value of *n*/*m*. Two datasets in (b) have the same number of measurements 1024, however, the MSE with 10000 voxels increases much later than the one with 1024 measurements.

In the offline preiteration step, the approximation of *A* ^+^ is obtained by the iteration of (7) with proper iterative number *K*. However, in the simulation the iterative method is found to have the semiconvergence property. This is probably due to the accumulated round-off error in the computation. So the optimal iteration number should be determined according to experience or prior information about the system. In our simulation, a pretest with a known distribution of fluorescence yield *X* is performed to choose *K* for the particular imaging system. And the mean squared error (MSE) between the original *X* and the reconstructed $\hat{X}$ is used as a criterion of the reconstructed quality. We investigated how the MSE changed against iteration times for several imaging systems with different sizes (*M* measurements and *N* voxels). Figure 2 shows that there is a relative flat segment where MSE changes very slowly before the iterative number begins to rise significantly. So the proper iteration number can be chosen in this iteration number range. Figure 2 is obtained in a noise-free environment. However, it is also found that when noise exists, the MSE rises earlier than in a noise-free system. For different levers of noise, the iteration numbers where MSE rises are different.

With the iterative result *S_k_* and the simulated detection *U*, the distribution of fluorescent yield can be well reconstructed simply by *X_k_* = *S_k_* *U*. In our simulation, *X_k_* is then modified by including a nonlinear function *f* to constrain the reconstructed values to [0, 0.05], that is

(14) $$\begin{array}{l} f\left(X_{k}\right)=\left\{ \begin{array}{l} 0,\;\;\;\;\;\;\;\;\;\;\;\;X_{k}\leq 0,\\ X_{k},\;\;\;\;\;\;\;\text{otherwise,}\\ \text{0}\text{.05,}\;\;\;\;\;X_{k}>0.05. \end{array}\right. \end{array}$$

**Figure 3:**
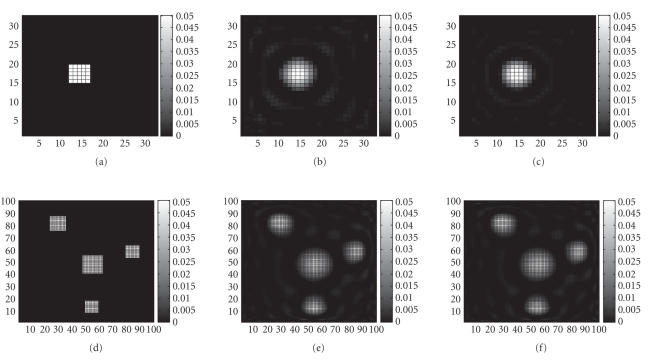
The original images and the reconstructions for single and multiprobe configurations with different datasets. (a) is the original distribution of the fluorescent yield *η* with 32 ∗ 32 voxels. (b) and (c) separately show the reconstruction of (a) with 256 measurements and 1024 measurements. (d) is the original *η* with image size of 100 ∗ 100 voxels. (e) and (f) show the reconstructions with 1024 measurements and 2048 measurements, respectively. For all of (b), (c), (e), (f), the iteration time in the preiteration step is 60.

In the simulation, occasions of single-probe as well as multiprobe are reconstructed for several different dataset sizes. It is proved that the distribution of the fluorescent yield *η* can be well estimated by the fast algorithm (Figure 3). It can be seen that the algorithm works well when the measurement number is much less then the reconstructed number.

For different imaging subjects, the weighted matrix *A* may need to be updated, so it would be desirable to know how the inversion time of the preiteration changes with different-sized datasets. According to the results of convergence of the iteration in Figure 2, 60-time iterations are chosen for all of the following datasets in order to compare the reconstructed timescales. The results are shown in Table 1. It can be inferred that for the same number of measurements *M*, the computing time is approximately proportional to the number of reconstructed voxels *N*. However, if *N* remains constant, when *M* rises to *l* · *M*, the computing time will increase to nearly *l* ^2^ times of the original.

## 5. DISCUSSION AND CONCLUSIONS

With the preiteration method, we have demonstrated reconstruction of fluorescence concentration by using simulation data based on the analytical model with first-order Born approximation. Although in this paper, the fast algorithm is simply demonstrated with the analytical solution for specific medium geometry, it could also be applied to arbitrary geometries, where the model in (1) is discretized by numerical methods [7, 10] or the Kirchhoff approximation [5].

In the simulation, a pretest should be done to determine the proper iteration number. A relationship between the convergence property and the dataset size is also obtained through the investigations and it can be found from Figure 2 that in noise-free environment, the number of iterations when the MSE begins to rise mainly depends on the ratio of the voxels number *N* and the number of measurements *M*, but not on the absolute value of them. This result will be helpful for the determination of the proper iteration number. For example, the convergence property of large dataset can be predicted from a smaller one with the same *N*/*M*. For a system with fixed measurement size, the larger the reconstructed mesh number is, the later will the MSE curve begin to rise.

The computation burden of the second-order iteration is further investigated in our work. It can be inferred from (7) that one iteration needs 2*M* ^2^ · *N* times multiplication. So the computation burden is proportional to the number of reconstructed points *N* when measurement number *M* stays unchanged and to *M* ^2^ when *N* is constant. This result is well proved in the simulation by the listed computing time for different numbers of measurements and voxels in Table 1.This feature should be very suitable for imaging systems where the number of voxels is always much larger than measurement number such as FODT. The results of both convergence property and computation burden indicate that the algorithm is very suitable for imaging systems in which the boundary measurement number is much less than the inside reconstructed voxels. In addition, the reconstructed images in Figure 3 showed that the algorithm works well for these kinds of system.

The most promising feature of the algorithm is the rapid reconstruction speed. It significantly accelerates the reconstruction process in the following two aspects. First, when the weighted matrix stays constant or the variance can be ignored, by allowing the time-consuming iteration to be performed offline, it provides great computational facility, which is just a unique matrix vector multiplication. Second, in the preiteration step, it is the second-order iteration form of (7) that exponentially improves the speed of the iterative process, which makes the algorithm feasible in practice and can be finally applied to FODT with datasets of large size. For example, to reconstruct the same quality images with 60 iterations of (7) (the reconstructed images are shown in Figure 3 and the computing time is shown in Table 1), it will cost about 2^60^ iterative steps using Landweber method or the first-order iterative form, requiring days for the reconstruction. So the first-order form is not practical for FODT of large-sized datasets even in the preiteration step. Therefore, the results demonstrate that the time efficiency of both the preiteration process and the online reconstruction is the most important advantage of the algorithm. It will be helpful to promote the development of real-time image reconstruction systems and dynamic monitoring of molecular activity.

## Figures and Tables

**Table 1:** Computation time for 60 iterations.

*m*	*n*		

	1024	4096	10000

256	4.2321 s	16.4102 s	39.2953 s

512	16.0307 s	61.5063 s	147.5493 s

1024	61.6572 s	238.5744 s	565.6728 s
